# Incidence of tick-borne spotted fever group *Rickettsia* species in rodents in two regions in Kazakhstan

**DOI:** 10.1038/s41598-022-19145-0

**Published:** 2022-09-01

**Authors:** E. Wagner, N. Tukhanova, A. Shin, N. Turebekov, Z. Shapiyeva, A. Shevtsov, T. Nurmakhanov, V. Sutyagin, A. Berdibekov, N. Maikanov, I. Lezdinsh, K. Freimüller, R. Ehmann, C. Ehrhardt, S. Essbauer, L. Peintner

**Affiliations:** 1 Section of Experimental Virology, Institute of Medical Microbiology, Jena University-Hospital, Jena, Germany; 2 Department Virology and Intracellular Agents, Bundeswehr Institute of Microbiology, German Centre for Infection Research, Munich Partner Site, Neuherbergstraße 11, 80937 Munich, Germany; 3 Center for International Health, University Hospital, LMU, Munich, Germany; 4 Aikimbayev’s National Scientific Center for Especially Dangerous Infections, Almaty, Kazakhstan; 5 Scientific Practical Center of Sanitary Epidemiological Expertise and Monitoring-Branch of the National Center for Public Health, Almaty, Kazakhstan; 6 Branch Aikimbayev’s National Scientific Center for Especially Dangerous Infections, Taldykorgan Antiplague Station, Taldykorgan, Kazakhstan; 7 Branch Aikimbayev’s National Scientific Center for Especially Dangerous Infections, Oral Antiplague Station, Oral, Kazakhstan

**Keywords:** Bacterial infection, Microbial ecology

## Abstract

Records on the distribution of *Rickettsia* spp. in their natural hosts in Central Asia are incomplete. Rodents and small mammals are potential natural reservoirs for *Rickettsiae* in their natural lifecycle. Studies about the maintenance of *Rickettsia* in wild animals are available for Western nations, but—to our knowledge—no studies and data are available in the Republic of Kazakhstan so far. The first case description of Rickettsioses in Kazakhstan was made in the 1950ies in the Almaty region and now Kyzylorda, East Kazakhstan, Pavlodar and North Kazakhstan are endemic areas. The existence of murine and endemic typhus was proven in arthropod vectors in the regions Kyzylorda and Almaty. Here we show for the first time investigations on tick-borne *Rickettsia* species detected by a pan-rickettsial citrate synthase gene (*gltA*) real-time PCR in ear lobes of small mammals (n = 624) in Kazakhstan. From all analysed small mammals 2.72% were positive for *Rickettsia raoultii, R. slovaca *or* R. conorii.* Sequencing of the rickettsial gene *OmpAIV* and the *23S–5S* interspacer region revealed a similar heritage of identified *Rickettsia* species that was observed in ticks in previous studies from the region. In summary, this study proves that rodents in Kazakhstan serve as a natural reservoir of *Rickettsia* spp.

## Introduction

*Rickettsiae* are small (0.3–0.5 by 0.8–2.0 µm) gram-negative intracellular bacteria, living in the cytosol of their host cells^1^. The genus *Rickettsia* is divided into four groups. (i) The spotted fever group (SFG) is linked predominantly with ticks and less often with fleas and mites, including Mediterranean-, Rocky Mountain- and Helvetica spotted fever. (ii) The typhus group (TG), which includes agents of epidemic typhus and murine typhus associated with lice and fleas. (iii) An ancestral group with *R. bellii* and *R. canadensis* and (iv) a transitional group with members of *R. akari* and *R. felis*.

The SFG, TG, and transitional groups include agents qualified to cause disease in human^1,2^. SFG is distributed worldwide and includes more than 30 species. At least 15 species cause disease, such as *R. rickettsii* in North America, which leads to Rocky Mountain spotted fever (RMSF) or *R. conorii,* causing Mediterranean spotted fever (MSF) in parts of Europe, Africa, and Asia^3–8^. From the TG *R. typhi* is causing murine/endemic typhus and more seldom *R. prowazekii* is inducing louse-borne or epidemic typhus in humans^3,9,10^. The transitional group comprises of three species transmitted by different vectors, among which *R. akari* is transmitted by mites (rickettsialpox), *R. australis* is transmitted by ticks (Queensland tick typhus), and *R. felis* is transmitted by fleas (flea-borne spotted fever)^8,11–16^.

Characteristic clinical manifestations caused by members of the SFG group include symptoms like fever, skin rash and in some cases also inoculation eschars. Moreover, other non-specific flu-like symptoms as febrile temperatures, cough, widespread lymphadenopathy, myalgia, abdominal ache and infections of the central nervous system are possible. Members of the TG group are causing epidemic typhus or murine typhus and come with symptoms such as high fever, headache and rashes on chest and extremities combined with nonspecific symptoms like cough, myalgia and malaise. In addition, neurological manifestations, like headache, meningitis and encephalitis, are also reported^2,17,18^.

Rickettsioses are generally distributed worldwide^4^. Sparse information is available on the disease and the distribution of tick-transmitted infections like Rickettsioses in Asian countries, but it is known that SFG and TG Rickettsia are present in Southeast Asia^2,3,18–20^. However, there are only incomplete records on the distribution of *Rickettsia* in Central Asia. In a representative country for the region, the Republic of Kazakhstan, most of the available information is based on anecdotal reports as described during an expedition by Bartoshevic to the region of Almaty in 1949–1951^21^. In 1961 clinical symptoms of tick-borne rickettsioses were observed in South Kazakhstan, West Kazakhstan, Pavlodar, North Kazakhstan and Akmola region^22^. In 1961, *R. sibirica* was detected in *Dermacentor marginatus* and *Haemaphysalis punctata* ticks collected from the Yenbekhikazakh district in Almaty region^23^. Other studies have confirmed, that *R. conorii* ssp. *caspia, R. slovaca, R. raoultii, R. aeschlimannii, R. asembonensis* and *R. felis* are circulating throughout Kazakhstan^17,24–30^.

Official endemic regions for Rickettsioses in Kazakhstan are currently North Kazakhstan, Pavlodar, East Kazakhstan and Kyzylorda. From 1995 to 2021 a total of 4627 human cases of tick-borne rickettsioses were reported in Kazakhstan. In recent years the incident rates in Kazakhstan increased from 0.41 (per 100,000 inhabitants per year) in 1995 to the highest rates of 1.19 in 2018 and 1.12 in 2019. The highest incidence seen from 1995 to 2021 was observed in 2019 in Kyzylorda region (incidence values of 1.64–12.68) and in Pavlodar region (incidence of 1.07–9.15)^31^. In comparison, in the USA 5000–6000 SFG cases were recorded during 2017, 2018 and 2019 with an incidence ranging between 1.5 and 1.8^32^.

While tick-associated Rickettsioses are monitored and reported in patients in Kazakhstan, relatively little is known about the spread of this zoonosis in the fauna of the country. A recent study on the prevalence of *Rickettsia* species in ticks in Almaty and Kyzylorda regions revealed a minimum infection rate (MIR) of 0.4–15.1% in Almaty region and 12.6–22.7% in Kyzylorda region. The detected species were *R. raoultii*, *R. slovaca,* a new *Candidatus R. yenbekshikazakhensis*, and the new genotype of *R. talgarensis*^33^.

Wild animals act as a natural reservoir for *Rickettsia* spp. and maintain the pathogens’ life cycle in nature^34,35^. Some data on the natural life cycle of *Rickettsia* are available from Europe, but no data from Central Asia are published so far. The European studies showed that screening ear pinnae of small mammals is a suitable tissue to detect *Rickettsia* species^36^.

The aim of this work was to identify *Rickettsia* spp*.* in ear pinnae of small mammals in West-Kazakhstan and Almaty region to study the distribution and the heritage of *rickettsial pathogens* in both regions.

## Material and methods

### Collection of tissues from small mammals

Small mammals trapping was conducted upon ethical approval of Kazakhstan local ethics committee at the National Scientific Center for Especially Dangerous Infectious in Almaty, Kazakhstan (protocol #4, 08.01.18) and the ethical committee of the Ludwig-Maximilians-University in Munich, Germany (opinion number 18-631) using snap traps in 2018 and 2019. Reporting of the animal experiments followed the recommendations in the ARRIVE guidelines. In West-Kazakhstan region, small mammals were trapped in 19 trapping sites of the four districts: Bayterek, Borili, Terekti, and Taskala. In Almaty region, small mammals were trapped in the three districts Tekeli, Rudnichniy, and Bakanas. In Almaty city small mammals were trapped in seven trapping sites (detailed location information see Supplementary Table 1 and Tukhanova et al*.*^37^). From the 624 trapped small mammals, ear pinnae were removed aseptically and stored in RNAlater (ThermoFisher Scientific, Waltham, United States), at − 20 °C. All methods were carried out in accordance with relevant guidelines and regulations.

### DNA extraction

Ear pinnae from small mammals were homogenized with two stainless steel beads and 1 ml of cell culture medium (Gibco™ MEM, ThermoFisher Scientific, Massachusetts, United States) using the TissueLyser II (2 min at 30 Hz) (Qiagen, Hilden, Germany). The homogenized samples were centrifuged for 5 min at 20,000×*g*.

DNA was isolated from 350 µl of the supernatant using QiAmp DNA Mini Kit (Qiagen, Hilden, Germany) according to the manufacturer’s instructions and stored in aliquots at − 20 °C.

### Real-time PCR approach

A real time PCR assay to screen for rickettsial DNA in the rodent ear pinnae was performed using the LightCyclerFastStart DNA Master HybProbe System (Roche, Basel, Switzerland) and a Rotor-GeneQ (Qiagen, Hilden, Germany) targeting the pan-rickettsial citrate synthase gene (*gltA*). An Uracil-DNA-glycosylase (UDG) incubation step was added to get rid of any carry-over PCR products between the reactions^36,38^. The total volume of the assay was 20 µl, incorporating 5 µl sample (containing up to 500 ng of DNA). The assay included 0.5 µM of primers PanRick_*gltA*_2 forward (5′-ATA GGA CAA CCG TTT ATT T-3′) and PanRick_*gltA*_2 reverse (5′-CAA ACA TCA TAT GCA GAA A-3′) and 0.2 µM of the probe PanRick_*gltA*_2_taq (5′-6FAM-CCT GAT AAT TCG TTA GAT TTT ACC G-DB-3′)^33,36,38^.

### Conventional PCR to generate DNA fragments for sequencing

Real-time PCR positive samples were further investigated using conventional PCR to amplify a fragment of the *outer membrane protein OmpAIV* (primers RR 190-5125: 5′-GCG GTT ACT TTA GCC AAA GG-3′, cRR 190-6013: 5′-TCT TCT GCG TTG CAT TAC CG-3′)^36,38,39^ and the *23S–5S* interspacer region (23S forward: 5′-GAT AGG TCG GGT GTG GAA GCA C-3′, 23S reverse: 5′-GGG ATG GGA TCG TGT GTT TCA C-3′)^40^ according to published protocols^33,36,38–40^ for subsequent sequencing. The total volume of the PCR assay was 50 µl with a final primer concentration of 0.5 µM and 5 µl of DNA sample (containing up to 500 ng of DNA). PCR products were analysed on agarose gels, with an expected band between 378–532 bp for the *23S–5S* interspacer region^40^ and 888 bp for the *OmpAIV*^39^.

### Sequencing

All conventional PCR products targeting the partial *OmpAIV* and *23S–5S* interspacer region were purified using the QIAquick PCR purification kit (Qiagen, Hilden, Germany). Sequencing was performed according to manufacturer’s instructions using a BigDye Terminator v3.1 Cycle Sequencing Kit (Applied Biosystems, Waltham, USA) and a 3730xl DNA Analyzer (Applied Biosystems, Waltham, USA).

### Phylogenetic analysis

Before BLAST-aided species determination and phylogenetic tree analysis the primer sequences were deleted from the sequences and then aligned in BioEdit 7.2.5^41^. Nucleotide sequence analyses were performed with Chromas Lite, version 2.1 (Technelysium Pty Ltd, South Brisbane, Australia) and compared for similarity to sequences deposited in NCBI GenBank. Phylogenetic trees were constructed in MEGA X with the Maximum Likelihood method based on the Tamura 3-parameter model^42^. Obtained *OmpAIV* and *23S–5S* interspacer nucleotide sequences were deposited in NCBI GenBank database under accession numbers ON604636–ON604650.

## Results

**Figure 1:**
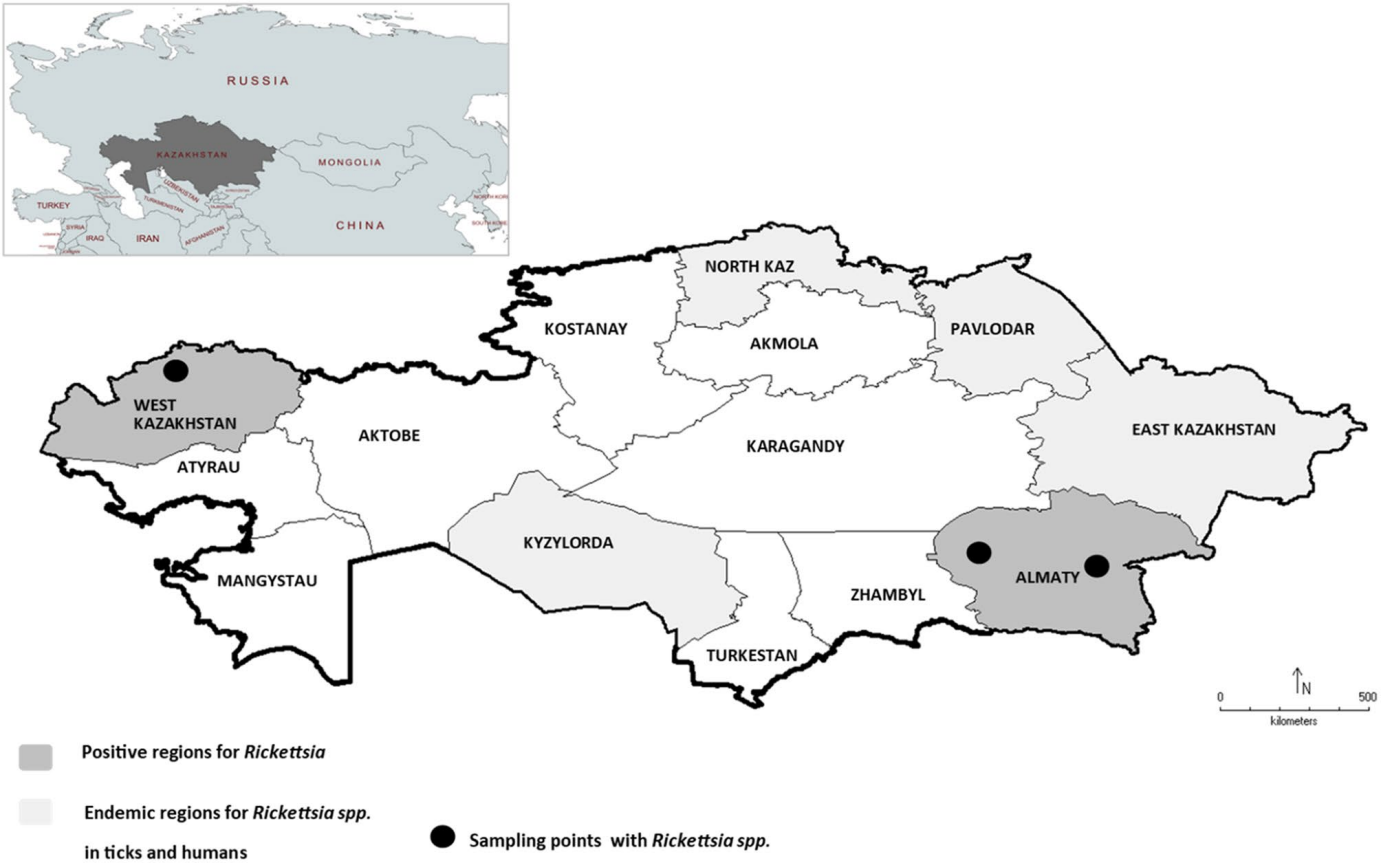
Investigation of *Rickettsia* spp. in Kazakhstan. Rickettsioses in humans are endemic in North Kazakhstan, Pavlodar, East Kazakhstan and Kyzylorda (light grey marked areas). Small mammals and rodents were investigated in West Kazakhstan and Almaty region (dark grey marked areas) with indicated positive sampling spots (•) in 2018 and 2019. In West-Kazakhstan in the area of Bayterek and in Almaty region in Tekeli (left •) and Bakanas (right •).

Eleven species of small mammals were collected from 29 trapping sites from West-Kazakhstan region, Almaty region and Almaty city (Fig. 1) in 2018 and 2019. The 624 small mammals were grouped into either rodents or insectivores. Members of the families *Cricetidae* (*Microtus arvalis* (n = 87), *Clethrionomys glareolus* (n = 13) and *Microtus kirgisorum* (n = 49)), of *Muridae* (*Apodemus uralensis* (n = 259), *Mus musculus* (n = 128), *Rattus norvegicus* (n = 39), *Meriones meridianus* (n = 2)) and of *Gliridae* (*Dryomys nitedula* (n = 15)) were examined. In addition, insectivores including *Crocidura suaveolens* (n = 28) and members of *Sorex* spp. (n = 4) (Supplementary Table 1).

From the 624 screened ear pinnae collected from small mammals, 17 (2.72%) were positive for the pan-*Rickettsia* citrate synthase gene *gltA* (Table 1). Rickettsia were detected in rodents captured in either the Bakanas district (Almaty region) from *M. musculus* (n = 8) and in *M. meridianus* (n = 1) or in the area of Tekeli from *M. arvalis* (n = 2) and *A. uralensis* (n = 1), 200 km to the east of Bakanas. In addition, in the West-Kazakhstan region five positive samples were detected from the Bayterek district, with a prevalence of 2.3% (n = 5/220) in *A. uralensis* (*n* = 4) and *M. arvalis* (n = 1).

**Table 1:** Results of *Rickettsia *spp*.* investigation. 624 small mammals from Almaty and West Kazakhstan region sampled in 2018 and 2019 have been first screened by a real-time PCR targeting the *citrate synthase gene* (*gltA*). Positive rodents have been further investigated by conventional PCR targeting a fragment in the *outer membrane protein* (*OmpAIV*) and the *23S–5S* interspacer region. Obtained sequences were deposited at GenBank (ON604639–ON604650). All gained sequences have been identified by BLAST and were compared in the phylogenetic trees of Figs. 2 and 3. AO = Akmola region, Tek = Tekeli, Bak = Bakanas, Bay = Bayterek, WKO = West Kazakhstan region, *M. arvalis* = *Microtus arvalis, M. musculus* = *Mus musculus, A. uralensis* = *Apodemus uralensis*, *R*. = *Rickettsia*, × = no sequence generated, *** = Fragment too short for phylogenetic analysis.

Region	Year	Sample ID	Rodent Species	*gltA*—citrate synthase realtime PCR	*OmpAIV*—outer membrane protein A			*23S–5S*—interspacer region		
					Sequence check by BLAST, *Rickettsia* species, GenBank ID, per Ident [%]	Species according Fig. 2	GenBankID	Sequence check by BLAST, *Rickettsia* species, GenBankID, per Ident [%]	Species according Fig. 3	GenBankID
Almaty region	2018	AO-Tek-2018_32	*M. arvalis*	Positive	×	×		×	×	
		AO-Tek-2018_34	*M. arvalis*	Positive	*R. raoultii* Isolate Tekeli 041, MG973997, 100%	*R. raoultii*	ON604636	×	×	
		AO-Bak-2018_1	*M. musculus*	Positive	*R. raoultii* Isolate Tekeli 041, MG973997, 100%	*R. raoultii*	ON604637	*R. raoultii*, MG450326, 100%	*R. raoultii*	ON604645
		AO-Bak-2018_2	*M. musculus*	Positive	*R. raoultii* strain Khabarovsk, AH015610, 100%	*R. raoultii*	ON604638	*R. raoultii* strain Khabarovsk, CP010969, 100%	*R. raoultii*	ON604646
		AO-Bak-2018_3	*M. musculus*	Positive	*R. raoultii* Isolate Tekeli 041, MG973997, 100%	*R. raoultii*	ON604639	*R. raoultii*, MG450326, 100%	*R. raoultii*	ON604647
		AO-Bak-2018_5	*M. musculus*	Positive	*R. raoultii* strain Khabarovsk, AH015610, 100%	*R. raoultii*	ON604640	*R. raoultii* strain Khabarovsk, CP010969, 100%	***	
		AO-Bak-2018_6	*M. musculus*	Positive	×	×		×	×	
		AO-Bak-2018_7	*M. musculus*	Positive	*R. raoultii* strain Khabarovsk, AH015610, 100%	*R. raoultii*	ON604641	*R. raoultii* strain Khabarovsk, CP010969, 99,6%	***	
		AO-Bak-2018_8	*M. musculus*	Positive	×	×		*R. raoultii* strain Khabarovsk, CP010969, 100%	***	
		AO-Bak-2018_13	*M. musculus*	Positive	*R. slovaca* Isolate Tekeli, MG973999, 100%	*R. slovaca*	ON604642	*R. raoultii* strain Tekeli, MG974041 100%	*R. raoultii*	ON604648
		AO-Bak-2018_14	*M. musculus*	Positive	×	×		×	×	
	2019	AO-Tek-2019_51	*A. uralensis*	Positive	×	×		×	×	
West Kazakhstan region	2018	WKO-Bay-2018_20	*A. uralensis*	Positive	×	×		*R. slovaca*, MG450332, 99,10%	*R. slovaca*	ON604649
		WKO-Bay-2018_23	*A. uralensis*	Positive	×	×		×	×	
		WKO-Bay-2018_26	*A. uralensis*	Positive	*R. raoultii* Isolate Tekeli 041, MG973997, 100%	*R. raoultii*	ON604643	×	×	
		WKO-Bay-2018_39	*M. arvalis*	Positive	*R. raoultii* Isolate Tekeli 041, MG973997, 100%	*R. raoultii*	ON604644	×	×	
	2019	WKO-Bay-2019_40	*A. uralensis*	Positive	×	×		*R. conorii* strain 1450, AY125012, 99%	*R. conorii*	ON604650

The prevalence of rickettsial infection in the different species varied depending on the region. In Almaty region the prevalence is 50% for *M. meridianus* (n = 1/2), 12% for *M. musculus* (n = 8/66), 2.7% for *M. arvalis* (n = 2/74), and 0.76% for *A. uralensis* (n = 1/131), whereas in West-Kazakhstan region the prevalence of rickettsial DNA is 3.13% in *A. uralensis* (n = 4/128) and 7.7% in *M. arvalis* (n = 1/13).

Of all 17 *gltA* real-time PCR positive rodents, conventional PCR for detecting a part of the *outer membrane protein OmpAIV* and of the *23S–5S* interspacer region was performed to gain more information about the exact species of *Rickettsia* detected. In total 18 sequences were obtained, nine partial *OmpAIV*-*,* and nine partial *23S–5S* interspacer region sequences. The partial *OmpAIV* sequences, all obtained in 2018, are from the districts Tekeli (n = 1, AO-Tek-2018-34) and Bakanas (n = 6, AO-Bak-2018-1, -2, -3, -5, -7 and -13) in Almaty region and from the Bayterek area (WKO-Bay-2018-26 and -39) in West Kazakhstan region.

The partial *23S–5S* interspacer fragments were from Bakanas- (Almaty region: AO-Bak-2018-1, -2, -3, -5, -7, -8, -13) and Bayterek districts (West Kazakhstan region: WKO-Bay-2018-20), all obtained in 2018 and one from 2019 (WKO-Bay-2019-40). Obtained sequences were compared to publicly available sequences deposited in the NCBI GenBank database using NCBI BLAST and *R. raoultii*, *R. slovaca*, or *R. conorii* were returned as the putative species detected in the ear lobes.

In six samples, both *OmpAIV* and *23S–5S* interspacer sequences were obtained from the same ear lobe (AO-Bak-2018-1, -2, -3, -5, -7 and -13). However, only four of them yielded sequence reads long enough for a reliable phylogenetic analysis. Two *23S–5S* interspacer sequence reads that were too short (AO-Bak-2018-5 and -7) were excluded from the phylogenetic analysis (Table 1, marked with ***).

Three of the four paired samples for both gene loci (*OmpAIV* and *23S–5S* interspacer region) were *R. raoultii* (AO-Bak-2018-1, -2 and -3). However, one of the paired samples (AO-Bak-2018-13), showed the closest phylogenetic relationship to different rickettsial species for *OmpAIV* and *23S–5S* interspacer region, respectively. The *23S–5S* interspacer fragment revealed a very high sequence similarity (100% identity; 340 of 340 nt identical) to a *R. raoultii* isolate from Tekeli (MG974041but the partial *OmpAIV* sequence clustered with a very high resemblance (100% identity; 715 of 715 nt identical) to a published *R. slovaca* sequence from Tekeli (MG973999)^33^.

**Figure 2:**
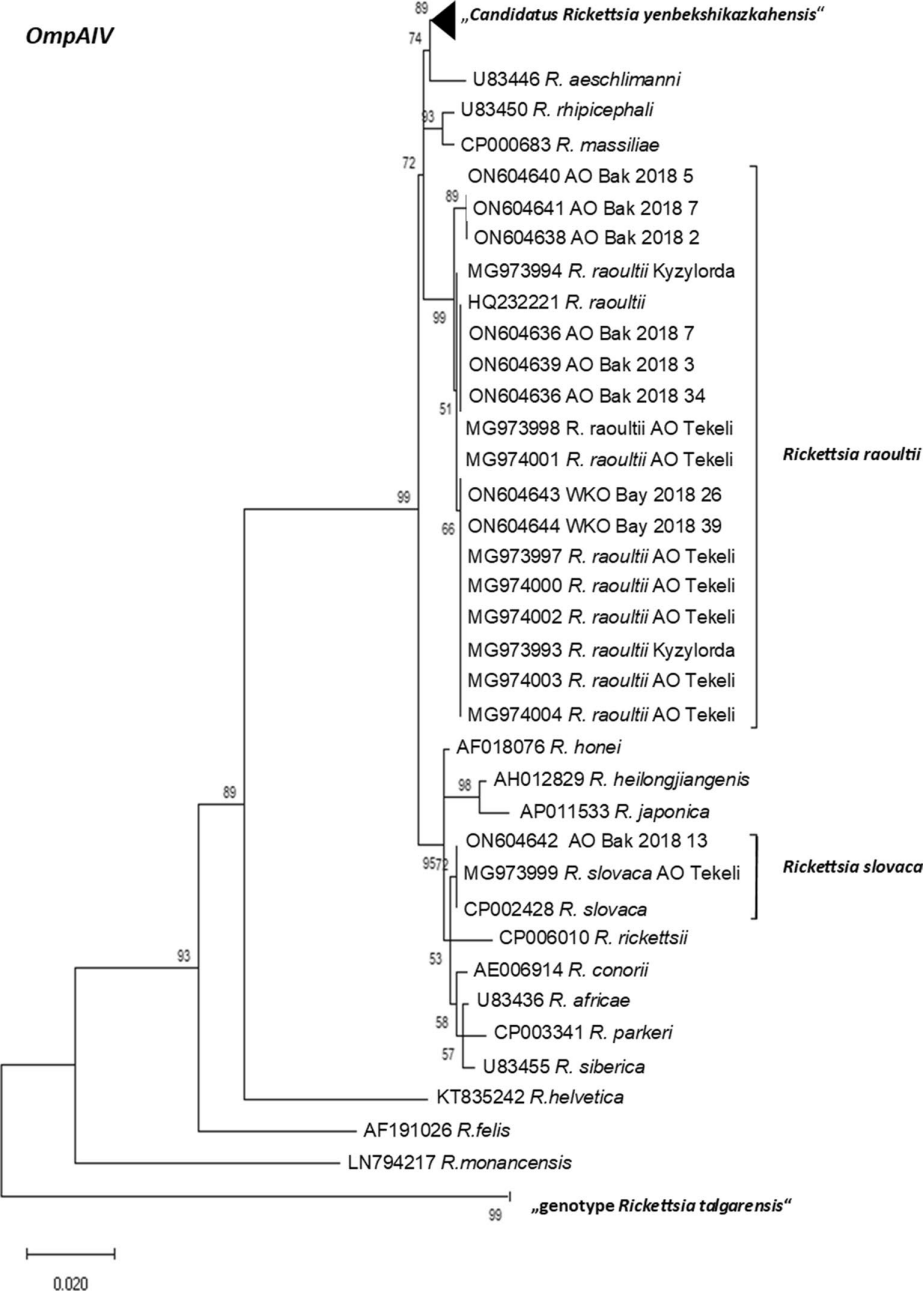
Maximum likelihood phylogenetic tree based on 68 partial *OmpAIV* DNA sequences. Nine sequences are originating from amplificates from small rodents from Kazakhstan and 59 from the GenBank database. Eight of the new generated sequences from Kazakhstan were 100% identical to *R. raoulti* and one were 100% identical to *R. slovaca*. In addition, 30 sequences form the *Candidatus Rickettsia yenbekshikazakhensis* and three sequences form the “genotype *Rickettsia talgarensis*” cluster. The tree with the highest log-likelihood (− 2445.21) is shown. There are in total 720 positions in the final dataset.

**Figure 3:**
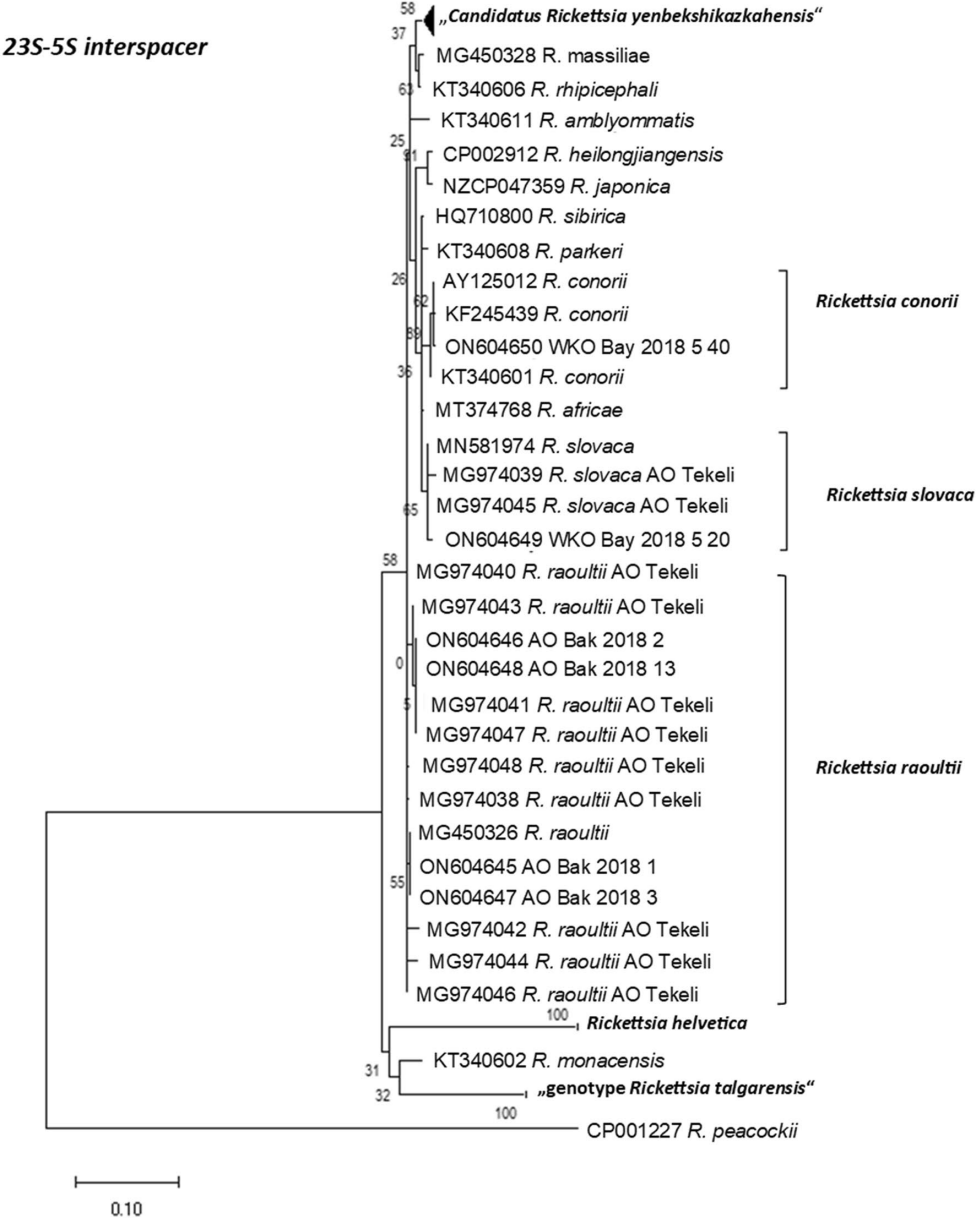
Maximum likelihood phylogenetic tree based on 46 partial 23S–25S interspacer DNA sequences. Six sequences originating from amplificates from small rodents from Kazakhstan and 40 from the GenBank database. Four of the new generated sequences from Kazakhstan were 90–100% identical to *R. raoulti*, one was 99% identical to *R. slovaca* and one was 99% identical to *R. conorii*. In addition, nine sequences form the *Candidatus Rickettsia yenbekshikazakhensis* and two sequences form the “genotype *Rickettsia talgarensis*” cluster. Three sequences form the cluster of *Rickettsia helvetica.* The tree with the highest log-likelihood (− 1639.61) is shown. There are in total 411 positions in the final dataset.

This ambiguity of the species can also be observed in the phylogenetic trees in Figs. 2 and 3, where representatives of worldwide distributed *Rickettsia* species and also published *Rickettsia* sequences from Kazakhstan like *R. raoultii* from Tekeli (Almaty region) and Kyzylorda region as well as the recently recorded “*Candidatus Rickettsia yenbekshikazakhensis*” and “*genotype Rickettsia talgarensis*”^33^ are included. AO-Bak-2018-13 clusters in Fig. 2 for *OmpAIV* with other strains of *R. slovaca* (MG973999 and CP002428) and in Fig. 3 for *23S–5S* interspacer region with representatives of *R. raoultii* from Tekeli (MG974041 and MG974047).

For some *gltA-*positive ear lobes only one sequence, either from *OmpAIV* or from the *23S–5S* interspacer region could be generated. Three partial sequences for *OmpAIV* (AO-Tek-2018-34, WKO-Bay-2018-26, and WKO-Bay-2018-39) showed high similarity to *R. raoultii* (MG973997 and AH015610, sequence identity 100%, Table 1). In addition, three individual sequences for the *23S–5S* interspacer region (AO-Bak-2018-8, WKO-Bay-2018-20, WKO-Bay-2019-40) clustered with *R. raoultii* (CP010969, sequence identity 99.7–100%)*, R. slovaca* (MG450332, sequence identity 99.44%) or *R. conorii* (AY125012, sequence identity 99.73%), respectively (Fig. 2 and Table 1).

## Discussion

To our knowledge, this study shows the first large-scale investigation of the prevalence of tick-transmitted *Rickettsia* in rodents in Kazakhstan. It is a follow-up study to recently published investigations on *Rickettsia* in ticks^33^ as well as agents for fever of unknown origin in patients^43^. Hence, it closes the gap between missing vector information and disease data in humans since it investigates the prevalence in natural hosts. Two regions of Kazakhstan were part of the study, the West-Kazakhstan region and the Almaty region in the south-east of the country including Almaty city. Both regions are not yet listed as endemic areas of rickettsiosis in Kazakhstan. Currently officially endemic areas for SFG rickettsioses in Kazakhstan are North-Kazakhstan, Pavlodar, East-Kazakhstan and Kyzylorda regions (Fig. 1). Only in these endemic areas the numbers of infections and incidences are recorded and listed in annual reports on case numbers^43,44^.

Here we show that *Rickettsia* species can be detected in the ear pinnae of several families of small mammals such as *Cricetidae* (*M. arvalis*) and *Muridae* (*M. musculus* and *A. uralensis*). From 624 screened small mammals, 17 were positive in a *gltA* screening PCR. This is a surprisingly high number given the fact that a screening based on PCR identifies only animals that have an acute infection with rickettsia. The rickettsial bacteraemia in rodents is rather short^45^, however, this is the critical phase for transmission to other ticks that might become infected while feeding. The rodents that yielded a positive *gltA* PCR such as *M. musculus* or *M. arvalis* are typical hosts of *Dermacentor marginatus*, a tick reported to carry *R. raoultii* and *R. slovaca* in previous studies in the investigated areas^46^. In comparison in Europe and Africa small mammals have a prevalence of *Rickettsia* spp. ranging from 5.2 to 17.6%, however those screenings were from areas that were suspected as rickettsia hotspots^36,47,48^. In our study randomly selected sampling spots were also included that had no previous history of rickettsioses.

To gain an idea on the genotypes circulating in small mammals in Kazakhstan we further amplified and sequenced two partial gene loci, *OmpAIV* and the *23S–5S* interspacer region, from the *gltA* positive samples. Of 17 positive ear pinnae we could retrieve six partial fragments of *23S–5S* interspacer regions and nine partial *OmpAIV* fragments. Using the NCBI BLAST nucleotide algorithm it was possible to gain more information on the genotype of the *Rickettsia* infecting the rodents. All identified sequences had high similarities to either *R. raoultii*, *R. slovaca, or R. conorii*. All three have been reported previously to reside in ticks in Kazakhstan^46^ and are of the SFG group. *R. slovaca* and *R. raoultii* are human pathogens that may cause scalp eschar and neck lymph adenopathy after a tick bite (SENLAT) syndrome that was also reported in Kazakhstan^44^.

A phylogenetic analysis of the obtained sequences with other sequences deposited in NCBI GenBank shows that the amplified fragments cluster closely with other rickettsia sequences that were obtained from ticks or small mammals in the region. Sequences from rodents in the Bakanas district had a close phylogenetic relationship to sequences obtained from ticks isolated in Tekeli, a city from the same region in Kazakhstan. In the Almaty oblast area *R. raoultii* is dominant and was mostly isolated from *Mus musculus*. Previous studies found *R. raoultii* in this region in *Dermacentor* ticks^33^, a tick that is known to feed on *M. musculus*. We are the first to sequence partial rickettsial genomes in the West Kazakhstan region, more than 2000 km to the west from the sampling sites in Almaty region. Still the phylogenetic distance is very short. This either proves that the genome of *Rickettsia* is highly evolutionarily conserved^49,50^, or allows the alternative explanation that the respective *Rickettsia* strains only recently moved to West Kazakhstan by migratory small mammals, birds or ticks they carry. This assumption may be supported by the fact that in Almaty region, the rate animals infected with rickettsia was about 3%, while in West Kazakhstan Oblast it was slightly lower at 2.2%.

In one ear lobe isolate (AO-Bak-2018-13) conflicting results were obtained from the *OmpAIV* and *23S–5S* interspacer sequencing. The *OmpAIV* returned as a *R. slovaca* and the *23S–5S* interspacer sequence grouped with *R. raoultii*. Theoretically it is possible, that this rodent was infected with two *Rickettsia* species at the same time. To explain this, it would be necessary to perform multi locus sequence typing (MLST) on seven or more loci of the rickettsia genome, howsoever this was not practical in the scope of this research project.

Unfortunately, not all positive *gltA* samples yielded amplicons for the *OmpAIV* or *23S–5S* interspacer region to obtain sequences for phylogeny. Other studies already showed that conventional PCR assays are less sensitive than real-time PCR assays^51^. This explains, why some lysates yielded positive results in the real time PCR but failed to produce an amplicon product in the conventional PCR.

The role of rodents and small mammals in the life-cycle of *Rickettsia* is far from being fully understood^8,36,52,53^. Ticks may transmit *Rickettsia* transovarially and also transstadially, which empowers the spread of the bacteria within the tick population without any additional vertebrate reservoir^54^. Co-feeding might serve also as a transmission route for *Rickettsia* spp.^55^. However, infection of vertebrates during tick feeding probably still plays a significant role. Indeed studies highlight that small animals—living in the wild or in laboratories—act as potential reservoir hosts for *Rickettsia* species^51,56,57^. Other studies, however, claim that rodents and small mammals do not carry any rickettsial DNA suggesting they do not play a role^53,58–61^. However, these findings should be taken with caution, as the selection of the organs examined and the capture sites may not have been optimal.

The ear lobes are a favourable region for ectoparasites like ticks and fleas that are feeding on rodents and other small mammals^36^. However, here we could not investigate whether rodents with *Rickettsia*-infected ears would also yield a positive PCR result when screening alternative organ tissues from the same animals. Other studies showed that rickettsial DNA can be detected in blood and skin biopsies (like ear pinnae), however with stark differences^47,51^. It is reported that the amount of rickettsial DNA in skin biopsies is threefold higher compared to the rickettsial DNA content of blood. Spleen samples have even lower DNA contents in infected animals^47^.

This screen for rickettsial DNA in small mammals and rodents completes other investigations on *Rickettsia* in Central Asia. A previous study in Kazakhstan on fever patients enrolled in Kyzylorda, an endemic region, and Almaty region, a non-endemic region, showed that in both regions 1.4% of 802 patients had acute SFG rickettsioses and 2.7% acute TG rickettsioses. A previous infection with SFG or TG rickettsia was detected in approximately 30% of the participating patients^43^. This study on patients was backed-up by a further investigation of ticks collected in the same regions (Almaty and Kyzylorda). Here, several *Rickettsia* species were identified in the arthropod vectors^33^. The MIR for rickettsia in the investigated ticks (*Dermacentor marginatus, D. reticulatus, Haemaphysalis punctata, Hyalomma asiaticum, *and* Rhipicephalus turanicus*) in Kyzylorda region was 12.6–22.7%, and in the non-endemic Almaty region 0.4–15.1%. In those ticks *R. raoultii* and *R. slovaca*, the new “*Candidatus R. yenbekshikazakhensis*” and a new genotype “*genotype R. talgarensis*” were detected^46^. The role of other vectors was assessed in additional studies. For instance, several *Rickettsia* species were detected in ticks and fleas collected all over Kazakhstan including Kyzylorda, East Kazakhstan, West-Kazakhstan and Almaty region^17,24–26,28,29^. At the Kazakhstan-China border in the Chinese province of Xinjiang several *Rickettsia* species (*R. raoultii, Candidatus R. barbariae* and genotype *Babesia*) were detected in *Haemaphysalis* ticks that were collected from *Vormela peregusna* (marbled polecats)^62^. These publications showed, that *Rickettsiae* are more widely distributed in Kazakhstan than officially reported and also reside in non-endemic areas such as the Almaty region. Moreover, microorganisms reside in dynamic borders and their prevalence in certain regions is heavily influenced by many factors such as climatic conditions, environmental changes, differences in urbanisation or land-use and other factors affecting both the bacteria themselves and their hosts. It is therefore essential to close the gaps in prevalence and vector data and keep a vigilant eye on changes. Continuous monitoring and surveillance are needed to keep track of any variations in these multi-faceted rickettsial ecosystems.

In summary this study highlights that rickettsial bacteria can be detected in small animals in non-endemic areas like Almaty region and West-Kazakhstan region. In areas where rickettsial infections are not monitored, the number of patients with rickettsiosis will be underestimated, as already postulated in a previous patient study in Almaty region^43^. Hence, physicians and policy makers in the Republic of Kazakhstan should be aware that rickettsioses are more widespread than previously thought.

## Supplementary Information

Supplementary Table 1.

## Data Availability

The data used and/or analysed during the current study are available from the corresponding author on reasonable request. All generated sequences were uploaded to NCBI Genbank and are accessible as ON604636, ON604637, ON604638, ON604639, ON604640, ON604641, ON604642, ON604643, ON604644, ON604645, ON604646, ON604647, ON604648, ON604649 and ON604650.
