# Supplementary material for: Incidence of tick-borne spotted fever group Rickettsia species in rodents in two regions in Kazakhstan
Source: Sci Rep. 2022 Sep 1;12:14872. doi: 10.1038/s41598-022-19145-0 (PMC9437098; doi:10.1038/s41598-022-19145-0)
Supplement: Supplementary file 1 — Supplementary Table 1. [file 41598_2022_19145_MOESM1_ESM.docx]

**Supplementary table 1:** Overview of collected small mammals, sampling sites, captured numbers and numbers of positive specimen.

| **Region** | **Year** | **sampling region** | **Sampling site** | **Rodents total** | **Family of *Cricetidae*** | | | **Family of *Muridae*** | | | | **Family of *Gliridae*** | **Insectivores** | | ***Rickettsia* positive of sampling site n (%): Realtime RT-PCR** | ***Rickettsia* in species** |
| --- | --- | --- | --- | --- | --- | --- | --- | --- | --- | --- | --- | --- | --- | --- | --- | --- |
|  |  |  |  |  | ***Mircrotus arvalis*** | ***Clethrionomys glareolus*** | ***Microtus kirgisorum*** | ***Apodemus uralensis*** | ***Mus musculus*** | ***Rattus norvegicus*** | ***Meriones meridianus*** | ***Dryomys nitedula*** | ***Crocidura suaveolens*** | ***Sorex spp.*** |  |  |
| **Almaty region** | 2018 | Almaty region | Bakanas | 15 | 0 | 0 | 0 | 0 | 13 | 0 | 2 | 0 | 0 | 0 | **9 (60)** | *M. musculus* (8)*;  M. meridianes* (1) |
|  |  |  | Tekeli | 75 | 14 | 0 | 0 | 41 | 8 | 0 | 0 | 11 | 0 | 1 | **2 (2.7)** | *M.arvalis* (2) |
|  |  | Almaty City | Flooplain small  Almatinka | 20 | 0 | 0 | 1 | 5 | 8 | 1 | 0 | 0 | 5 | 0 | 0 |  |
|  |  |  | Stroikombinat | 12 | 1 | 0 | 3 | 0 | 3 | 1 | 0 | 0 | 4 | 0 | 0 |  |
|  |  |  | Railway | 9 | 0 | 0 | 1 | 5 | 0 | 0 | 0 | 0 | 3 | 0 | 0 |  |
|  |  |  | Airport | 27 | 0 | 0 | 0 | 5 | 2 | 12 | 0 | 0 | 8 | 0 | 0 |  |
|  |  |  | Mercur | 12 | 0 | 0 | 0 | 9 | 0 | 0 | 0 | 0 | 3 | 0 | 0 |  |
|  |  |  | Autopark | 7 | 0 | 0 | 0 | 6 | 0 | 0 | 0 | 0 | 1 | 0 | 0 |  |
|  | 2019 | Almaty region | Tekeli | 80 | 29 | 0 | 0 | 43 | 6 | 0 | 0 | 2 | 0 | 0 | **1 (1.3)** | *A. uralensis* (1) |
|  |  |  | Rudnichniy | 30 | 30 | 0 | 0 | 0 | 0 | 0 | 0 | 0 | 0 | 0 | 0 |  |
|  |  | Almaty City | Airport | 35 | 0 | 0 | 2 | 5 | 6 | 19 | 0 | 0 | 3 | 0 | 0 |  |
|  |  |  | Railway | 39 | 0 | 0 | 22 | 6 | 9 | 0 | 0 | 0 | 1 | 1 | 0 |  |
|  |  |  | Mercur | 14 | 0 | 0 | 0 | 2 | 6 | 6 | 0 | 0 | 0 | 0 | 0 |  |
|  |  |  | Tropinka | 29 | 0 | 0 | 20 | 4 | 5 | 0 | 0 | 0 | 0 | 0 | 0 |  |
| **West-Kazakhstan region** | 2018 | WKO | Bayterek | 58 | 7 | 3 | 0 | 45 | 0 | 0 | 0 | 2 | 0 | 1 | **4 (10)** | *A. uralensis*(3);  *M. arvalis* (1) |
|  |  |  | Borili | 10 | 0 | 0 | 0 | 0 | 10 | 0 | 0 | 0 | 0 | 0 | 0 |  |
|  |  |  | Oral | 27 | 1 | 0 | 0 | 26 | 0 | 0 | 0 | 0 | 0 | 0 | 0 |  |
|  |  |  | Taskala District | 16 | 0 | 0 | 0 | 0 | 16 | 0 | 0 | 0 | 0 | 0 | 0 |  |
|  |  |  | Teretki | 9 | 0 | 9 | 0 | 0 | 0 | 0 | 0 | 0 | 0 | 0 | 0 |  |
|  | 2019 | WKO | Bayterek | 48 | 0 | 1 | 0 | 36 | 10 | 0 | 0 | 0 | 0 | 1 | **1 (2.1)** | *A. uralensis* (1) |
|  |  |  | Borili district | 15 | 0 | 0 | 0 | 5 | 10 | 0 | 0 | 0 | 0 | 0 | 0 |  |
|  |  |  | Oral | 10 | 0 | 0 | 0 | 10 | 0 | 0 | 0 | 0 | 0 | 0 | 0 |  |
|  |  |  | Teretki | 27 | 5 | 0 | 0 | 6 | 16 | 0 | 0 | 0 | 0 | 0 | 0 |  |
|  |  |  | **Total** | **624** | **87** | **13** | **49** | **259** | **128** | **39** | **2** | **15** | **28** | **4** | **17 (2.72)** |  |
